# Ultrasound-Assisted Extraction of Polysaccharides from Mulberry Leaves Using Response Surface Methodology: Purification and Component Identification of Extract

**DOI:** 10.3390/molecules30081747

**Published:** 2025-04-14

**Authors:** Teng Wang, Xiaolin Zou, Hong Zhang, Jiwen Li, Xiaoming Peng, Ruijun Ju, Zhaojun Jia, Zhenguo Wen, Cuiqing Li

**Affiliations:** 1 Department of Pharmaceutics, Beijing Institute of Petrochemical Technology, Beijing 102627, China; 2023520321@bipt.edu.cn (X.Z.); zhanghong@bipt.edu.cn (H.Z.); lijiwen@hebau.edu.cn (J.L.); pengxiaoming@bipt.edu.cn (X.P.); juruijun@bipt.edu.cn (R.J.); 0020200029@bipt.edu.cn (Z.J.); wenzhenguo@bipt.edu.cn (Z.W.); licuiqing@bipt.edu.cn (C.L.); 2 College of Science and Technology, Hebei Agricultural University, Huanghua 061100, China

**Keywords:** polysaccharides, ultrasound, mulberry leaves, Box–Behnken

## Abstract

In this study, an ultrasonic-assisted procedure for the extraction of mulberry leaf polysaccharides (MLPs) was investigated using response surface methodology with a 29-run Box–Behnken design. Four factors were investigated, and it was found that the factors influencing the process, in order of significance, were the extraction temperature > liquid-to-material ratio > ultrasonic power. Considering practical conditions, the parameters were adjusted to a liquid-to-material ratio of 16:1 mL/g, extraction time of 58 min, extraction temperature of 65 °C, and ultrasonic power of 500 W. Under these conditions, the yield of MLPs was 14.47%, which is close to the predicted value, indicating that the extraction process optimized by response surface methodology (RSM) is feasible. The separation and purification effects of macroporous resin and activated carbon on MLPs were investigated, with the D152 resin being considered the most suitable choice. The optimal separation conditions were found to be a sample concentration of 0.5 g/mL and an optimal flow rate of 1 mL/min. Thin-layer chromatography and infrared spectroscopy revealed that polysaccharides extracted from mulberry leaves are primarily composed of rhamnose, xylose, and arabinose. In conclusion, this study successfully optimized the ultrasonic-assisted extraction process of MLPs through response surface methodology, determined the optimal parameter combination, and verified its efficiency and stability. Under the optimal conditions obtained for ultrasonic-assisted extraction, the yield of MLPs is significantly higher than that reported in the previous literature. The decolorization process of crude mulberry leaf polysaccharide extract was also investigated, and the purified MLPs have clear monosaccharide composition and structural characteristics, providing a theoretical basis and technical support for their application in functional food or drug development.

## 1. Introduction

*Morus alba* L., commonly known as mulberry, is a woody plant within the genus Morus of the Moraceae family. It is widely distributed across China, and its leaves, root bark, and branches have traditionally been employed in Chinese medicine for the treatment of fever, liver protection, vision improvement, urine promotion, and blood pressure reduction [1,2]. The mulberry leaf has been recognized as a resource with dual properties of both food and medicine, as indicated by its inclusion in the list of homologous resources issued by the National Health Commission of China. Furthermore, it is documented in the latest edition of the “Pharmacopoeia of the People’s Republic of China” (2025 edition). Due to its remarkable biological properties and nutritional content, the mulberry leaf is widely employed as a premium medicinal resource and a key ingredient in the development of functional foods in China, Japan, South Korea, and numerous other nations. Modern scientific investigations have demonstrated that mulberry leaves are rich in a variety of nutrients, including proteins, carbohydrates, vitamins, organic acids, and trace elements. Additionally, they also possess many bioactive constituents, such as polysaccharides, flavonoids, alkaloids, and amino acids. Among these, mulberry leaf polysaccharides (MLPs) have garnered considerable interest due to their diverse physiological benefits. In recent years, extensive research has highlighted the potential medicinal value of MLPs, demonstrating their potent biological activities, including hypoglycemic, antihypertensive, anti-hyperlipidemia, antioxidant, immunomodulatory, antibacterial, and antiatherosclerotic effects. Furthermore, MLPs have been found to be safe and effective with low toxicity and minimal side effects [3,4,5].

The commonly used extraction processes for MLPs include solvent extraction, microwave-assisted extraction, and ultrasound-assisted extraction, among others. Different extraction methodologies result in variations in the yield of MLPs. Additionally, the yield of polysaccharides may fluctuate even when the same extraction approach is utilized, which can be ascribed to differences in the source of the mulberry leaves, process variables, and the analytical techniques employed for assessing the polysaccharide content [5]. Conventional solvent extraction (CSE) is the most widely used method for extracting polysaccharides. While CSE is straightforward and safe, the high temperatures and extended extraction times associated with it can result in the degradation of polysaccharides and a reduction in their pharmacological activity [6,7]. In recent years, ultrasound-assisted extraction (UAE) methods have been used for the extraction of polysaccharides from various natural plants. The acoustic cavitation in UAE leads to the disruption of cell walls, a decrease in particle size, and an improvement in the contact between solvents and target compounds [8,9,10]. It has the advantages of wide applicability, good reproducibility, high selectivity, and short operation time. Due to its lower energy consumption, reduced solvent usage, higher extraction efficiency, and greater level of automation, UAE is considered preferable over CSE. To achieve an optimal yield, these extraction methods should be optimized.

The MLPs obtained by extraction are crude polysaccharides, which contain many impurities, such as pigments, inorganic salts, proteins, and low-molecular-weight nonpolar substances, which need to be further separated and purified. Among these impurities, the presence of pigments poses the greatest challenge for the further application of MLPs in the food and pharmaceutical industries. The coexisting pigments may complicate the procedures of separation, purification, and structural elucidation, which could adversely affect the biological activity of the MLPs. Consequently, decolorization is acknowledged as an essential step in the preparation of MLPs. Decolorization is typically achieved using materials or reagents like H_2_O_2_, activated carbon, microporous adsorption resin, etc. [11].

The objective of this study was to optimize the conditions for the ultrasonic extraction of polysaccharides from mulberry leaves using response surface methodology (RSM). This approach enables the collective optimization of all influencing parameters, thereby overcoming the limitations inherent in single-factor optimization processes [12,13,14]. A four-variable Box–Behnken design with 29 experiments was implemented to identify significant factors affecting the extraction yield and to establish the optimal conditions for maximum extraction efficiency. The separation and purification effects of MLPs were also investigated with activated carbon and eight macroporous resins, each varying in chemical and physical properties, to identify the optimum resin based on the decolorization ratio and recovery ratio. The purified component was characterized by thin-layer chromatography and infrared spectroscopy. This study systematically optimized the process conditions for the extraction and purification of polysaccharides from mulberry leaves, and offers a theoretical foundation for the extensive development and application of mulberry leaf polysaccharides.

## 2. Results

### 2.1. Effect of Factors on Extraction

#### 2.1.1. Effect of Liquid-to-Material Ratio on Yield of MLPs

**Figure 1:**
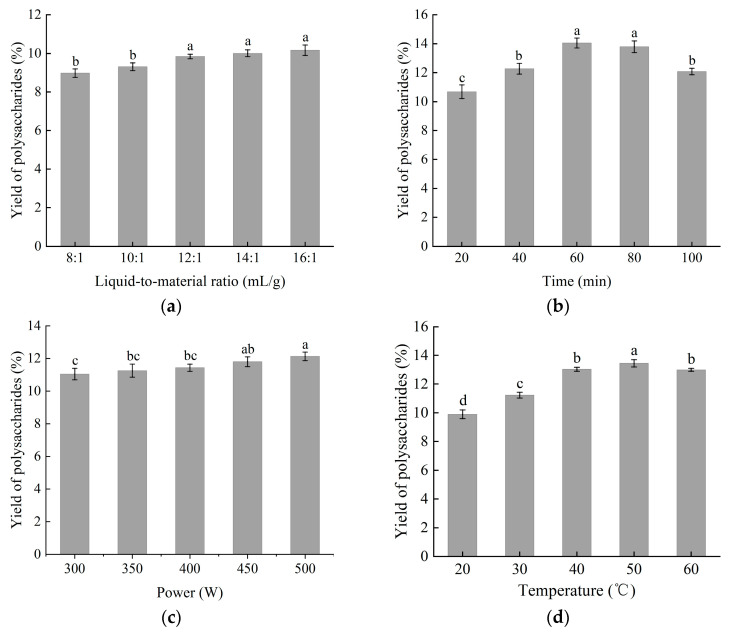
The effect of extraction parameters on the yield of polysaccharides. (**a**) Liquid-to-material ratio; (**b**) extraction time; (**c**) ultrasonic power; (**d**) extraction temperature. The values are expressed as means ± SD (n = 3). Different lowercase letters (a, b, c, and d) in the same figure indicate statistically significant differences (*p* < 0.05).

The yield of polysaccharides extracted using varying liquid-to-material ratios, ranging from 8:1 to 16:1, is depicted in Figure 1a. The extraction temperature and time were maintained at 50 °C and 1 h, respectively, while the ratio was increased from 1:8 to 1:16. A higher liquid-to-material ratio indicates a greater concentration gradient between the interior of the plant cells and the external solvent, facilitating the rapid diffusion of polysaccharides [15]. However, an excessively high liquid-to-material ratio extends the diffusion distance to the interior tissues. Consequently, the yield increased only modestly when the ratio was increased from 12:1 (mL/g). To prevent excessive solvent usage and to facilitate easier handling in subsequent processes, a ratio range of 1:12 to 1:16 was selected for further study using the Box–Behnken design.

#### 2.1.2. Effect of Extraction Time on Yield of MLPs

Extraction time is another factor that influences the yield of polysaccharides. As the extraction time was extended from 20 to 100 min, the results indicated that the extraction yield varied between 10.68% and 14.06%, as depicted in Figure 1b. Ultrasound assisted in releasing the polysaccharides from within the cells to the external solvent. The majority of the polysaccharides from broken cells were released during the initial stage of extraction [16]. However, a prolonged extraction time led to the degradation of polysaccharides. Consequently, the yield of polysaccharides continued to rise until the extraction time reached 60 min before it started to decline. Therefore, a time range of 40–80 min was determined to be optimal for the current experiment and was further employed in response surface methodology (RSM).

#### 2.1.3. Effect of Ultrasonic Power on Extraction Yield of MLPs

In this study, the influence of varying ultrasonic power on the extraction yield was investigated. The findings are presented in Figure 1c. The ultrasonic power was adjusted within a range of 300 to 500 W, while all other extraction parameters were kept constant. The results demonstrated that the extraction yield of polysaccharides increased concomitantly with the enhancement of ultrasonic power. This trend can be attributed to two primary physical phenomena associated with UAE: acoustic cavitation and diffusion through cell walls [17]. The application of ultrasonic energy facilitated the breakdown of cell walls, thereby accelerating the dissolution of polysaccharides. Consequently, a higher yield of polysaccharides was achieved with greater extraction power. However, acoustic cavitation may lead to the production of hydroxyl radicals, leading to chemical decomposition in extraction [18]. More chemical decomposition occurred with a stronger extraction power. Taking all the above factors into consideration, the power range of 400–500 W was identified as optimal for this extraction process and was subsequently employed for further optimization using response surface methodology (RSM).

#### 2.1.4. Effect of Extraction Temperature on Yield of MLPs

Figure 1d illustrates the effect of temperature on the extraction yield of polysaccharides. The temperature was varied from 20 to 60 °C. The extraction yield of polysaccharides was observed to increase with temperature from 20 to 50 °C, after which the yield started to decline with further temperature increments. This result is related to two main physical phenomena in UAE: acoustic cavitation and diffusion through the cell walls. The two phenomena were significantly enhanced by the extraction temperature. Therefore, the yield of polysaccharides increased with a higher temperature. However, a high temperature led to a decrease in surface tension and increase in vapor pressure within micro bubbles, causing the damping of the ultrasonic wave [19]. Thus, the yield of polysaccharides decreased when the extraction temperature was over 50 °C. Consequently, based on these findings, a temperature range of 30 to 50 °C was determined to be optimal for the extraction process and was chosen for subsequent optimization using response surface methodology (RSM).

### 2.2. Box–Behnken Design

#### 2.2.1. Model Building and Statistical Analysis

Based on the single-parameter studies, a Box–Behnken design was employed to investigate the extraction process under the following conditions: a liquid-to-material ratio ranging from 12:1 to 16:1, an extraction time ranging from 40 to 80 min, an ultrasonic power ranging from 400 to 500 W, and an extraction temperature ranging from 30 to 50 °C. As depicted in Table 1, a total of 29 experimental runs were conducted to optimize the three independent parameters in the current Box–Behnken design (BBD). These parameters were designated as Y, the response, specifically the yield of polysaccharides; X_1_, the liquid-to-material ratio (12:1–16:1); X_2_, extraction time (40–80 min); X_3_, ultrasonic power (400–500 W); and X_4_, extraction temperature (30–50 °C). Multiple regression analysis was applied to the experimental data to relate the response variable to the test variables through the following second-order polynomial Equation (1):

Y = 13.95 + 1.06 X_1_ + 0.2 X_2_ + 10.97 X_3_ + 3.23 X_4_ − 0.84 X_1_ X_2_ + 0.93 X_1_ X_3_ + 0.89 X_1_ X_4_ + 0.54 X_2_ X_3_ + 0.12 X_2_ X_4_ − 0.20 X_3_ X_4_ − 1.42 X_1_^2^ − 0.72 X_2_^2^ − 1.16 X_3_^2^ − 0.80 X_4_^2^ (1)

The significance of each coefficient in the model was assessed using the F-test and corresponding *p*-values, as presented in Table 2. The *p*-value served as a metric to determine the significance of each coefficient, thereby indicating the nature of the interactions among variables. An analysis of variance (ANOVA) for the quadratic regression model revealed that the model was highly significant, as indicated by the F-test with a very low probability value (*p* = 0.001). The model F-value of 56.47 suggested that the model was statistically significant, with only a 0.01% likelihood that such a large “Model F-value” could be attributed to random noise. The “Lack of Fit F-value” of 28.27 indicated that the lack of fit was significant, with a probability of only 0.28% that this value could be due to noise [20].

In Table 2, it is evident that the linear coefficients (X_1_, X_3_, X_4_), the quadratic term coefficients (X_1_^2^, X_2_^2^, X_3_^2^, X_4_^2^), and the interaction coefficients (X_1_X_2_, X_1_X_3_, X_1_X_4_, X_2_X_3_) are statistically significant. “Prob > F” values less than 0.05 denote that the model terms are significant. In this study, X_1_, X_3_, X_4_, X_1_^2^, and X_3_^2^ were identified as significant model terms with very small *p*-values (*p* < 0.0001). Additionally, X_1_X_2_, X_1_X_3_, X_1_X_4_, X_2_X_3_, X_2_^2^, and X_4_^2^ were determined to be important factors with small *p*-values (*p* < 0.01). Through a comparison of the F-values of each factor in Table 2, it can also be found that the factor X_4_ in this regression model has the most significant effect on the extraction rate of polysaccharides, followed by factors X_1_ and X_3_, and the significance order is as follows: extraction temperature (X_4_) > liquid-to-material ratio (X_1_) > ultrasonic power (X_3_) [21].

The coefficient of determination (R^2^ = 0.9827), the adjusted coefficient of determination (R^2^*_adj_* = 0.9652), and the coefficient of variation (C.V. = 4.84%) are presented in Table 3. These metrics suggest that the polynomial model exhibits high accuracy and good predictability. The predicted R^2^ (R^2^*_pred_* = 0.9008) is in reasonable agreement with the adjusted R^2^ (R^2^*_adj_* = 0.9562), indicating the model’s reliability. “Adeq. Precision” serves as a measure of the signal-to-noise ratio, with a value greater than 4 typically considered desirable. The “Adeq. Precision” value of 27.242 indicates that the model is sufficiently precise to navigate the design space effectively.

#### 2.2.2. Optimization of the Procedure

**Figure 2:**
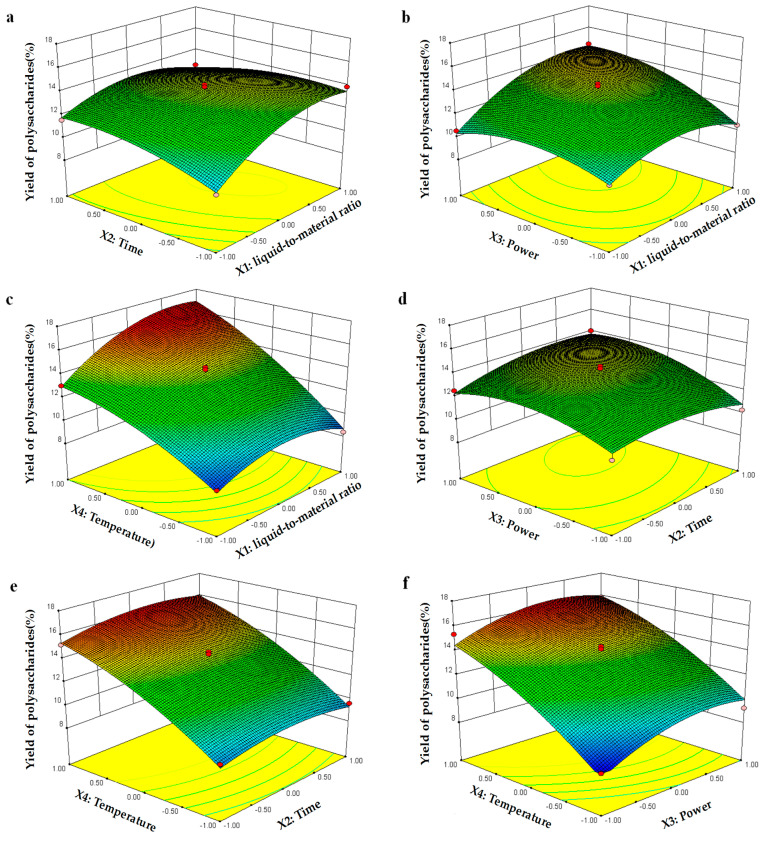
Response surface plots for interaction between various factors in yield of polysaccharides. (**a**) The two changed variables were liquid-to-material ratio and extraction time; (**b**) liquid-to-material ratio and ultrasonic power; (**c**) liquid-to-material ratio and extraction temperature; (**d**) extraction time and ultrasound power; (**e**) extraction time and extraction temperature; (**f**) ultrasonic power and extraction temperature. The color change in the figure from blue to green to red represents the change in polysaccharide yield from low to high. The faster the change, the steeper the slope, indicating a more significant impact on the experimental results.

Figure 2 presents the 3D response surfaces as graphical representations of the regression equation. These visual tools facilitate the interpretation of the relationship between the response variables and the experimental levels of each variable, as well as the nature of the interactions between the two test variables. The contour plot shapes, whether circular or elliptical, indicate the significance of the interactions between variables: circular shapes suggest negligible interactions, whereas elliptical shapes indicate significant interactions [22]. In the 3D response surface generated by the model for polysaccharide yield, the relationships between two variables are depicted on a 3D surface plot while the other variables are held at the zero level.

Figure 2a demonstrates that when X_3_ and X_4_ are fixed at the zero level, X_1_ and X_2_ exhibit a reciprocal interaction effect on the extraction yield. Specifically, when X_2_ is maintained at a lower level, the yield of polysaccharides increases with the increment in X_1_. That is, in the early stage of polysaccharide extraction, the yield significantly increases with the increase in the liquid-to-material ratio while other factors are fixed. Figure 2b illustrates that when X_2_ and X_4_ are fixed at the zero level, X_1_ and X_3_ display quadratic effects on the extraction yield. The yield initially increases and then decreases with the increase in X_1_ when X_3_ is kept at a lower level. This indicates that at low ultrasonic intensities, with other parameters held constant, the polysaccharide yield increases first and then decreases as the liquid-to-solid ratio increases. Figure 2c shows the 3D response surface plot and contour plot for varying X_1_ and X_4_ values, with X_2_ and X_3_ held at the zero level. These plots indicate that the extraction yield is significantly influenced by X_1_ and X_4_, which means that the liquid-to-solid ratio and temperature are the most crucial factors affecting the polysaccharide yield. This is consistent with the information reflected by the model F-value. Figure 2d reveals that X_2_ and X_3_ exhibit quadratic effects on the yield of polysaccharides when the other two variables are fixed at the zero level; however, these effects are not significant. The results presented in Figure 2e,f are consistent, and they indicate that when X_1_ and X_3_ are fixed at the zero level, the yield of polysaccharides significantly increases with the increment in X_2_ and X_4_. The same trend is reflected when X_1_ and X_2_ are fixed at the zero level, and the polysaccharide yield also significantly increases with the increment in X_3_ and X_4_. These results again indicate that the extraction temperature is the most important factor affecting the polysaccharide yield.

#### 2.2.3. Validation of the Model

Based on the data presented in Figure 2, the optimal extraction conditions for MLPs were determined to be a liquid-to-material ratio of 16.1:1 mL/g, an extraction time of 58.2 min, an ultrasonic power of 484.5 W, and an extraction temperature of 65.1 °C. The theoretical extraction yield of polysaccharides predicted under these optimized conditions was 15.17%. To validate the optimization, a confirmatory experiment was performed under slightly modified conditions: a liquid-to-material ratio of 16:1 mL/g, an extraction time of 58 min, an ultrasonic power of 500 W, and an extraction temperature of 65 °C. The actual yield obtained from the experimental set was 14.47 ± 0.76% (n = 5), which was in good agreement with the predicted value of the model equation (Table 4). This confirmed the adequacy of the response model for the optimization process.

Chen et al. [5] provided a review of past research on the extraction, separation, structural identification, and biological activities of MLPs conducted by various investigators in 2024. The method that yielded the highest MLP extraction rate using hot water extraction is reported by Samavati et al. [23], as summarized in the review. The method employed a liquid-to-material ratio of 18:1, an extraction temperature of 85 °C, and an extraction time of 300 min. Under these conditions, the yield of MLPs was 12.00 ± 0.42%. The method that yielded the highest MLP extraction rate using microwave-assisted extraction was reported by Thirugnanasambandham et al. [24]. The method employed a liquid-to-material ratio of 20:1, an extraction temperature of 88 °C, an extraction time of 11 min, and a microwave intensity of 170 W. Under these conditions, the yield of mulberry leaf polysaccharides was 9.41%. There have also been reports of the ultrasonic-assisted extraction of MLPs, with the highest yield reported by Ying et al. [25]. The method used involved a liquid-to-material ratio of 15:1, an extraction temperature of 80 °C, an extraction time of 20 min, and an ultrasonic intensity of 60 W. Under these conditions, the yield of MLPs was 10.79%. This study also examined the extraction efficiency of MLPs without the use of ultrasound assistance, while maintaining other optimal extraction conditions (method described in Section 3.3), resulting in an extraction yield of 10.88 ± 0.22%.

Compared with the aforementioned literature, the ultrasonic-assisted extraction method for MLPs proposed in this study has the highest polysaccharide yield to date. At the same time, it significantly reduces the extraction time compared to the hot water extraction method reported by Samavati. Compared to the microwave-assisted extraction method reported by Thirugnanasambandham and the ultrasonic-assisted extraction method reported by Ying, it significantly lowers the extraction temperature, effectively preventing the hydrolysis of polysaccharides, thereby increasing the yield of polysaccharides.

### 2.3. Purification and Separation of Polysaccharides by Macroporous Resin

#### 2.3.1. Resin Screening

Macroporous resins are specialized organic polymers synthesized via polycondensation or polymerization reactions. They are distinguished by their extensive pore structure and substantial surface area. The microporous resin is notable for its exceptional stability, high processing efficiency, cost-effectiveness, recyclability, and user-friendly operation, which positions it as a preferred technique for the decolorization of polysaccharides in academic and industrial settings [11].

**Figure 3:**
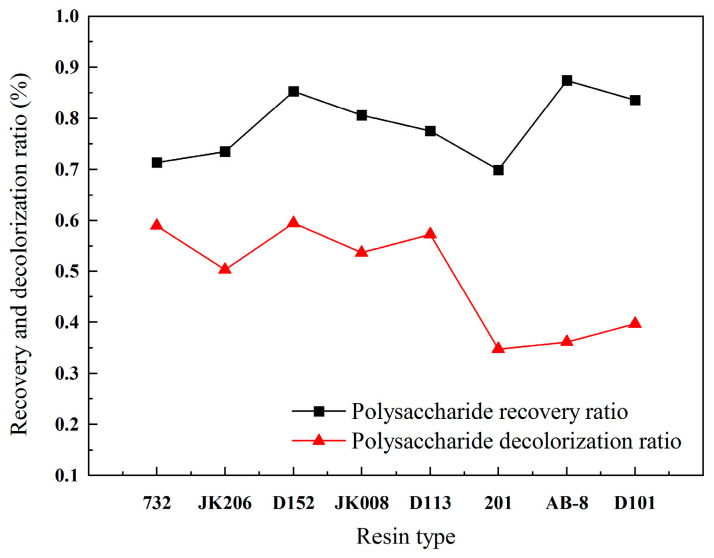
Recovery ratio and decolorization ratio of polysaccharides with different resins.

The efficacy of decolorization is intricately associated with the external and internal surface areas, pore dimensions, and the functional groups present in the resin. To achieve effective decolorization, the resin must demonstrate a high capacity for pigment adsorption, as well as the capability to recover polysaccharides. The criteria for selecting the optimal resin for decolorization encompass the evaluation of both the decolorization efficiency and the recovery yield. Figure 3 displays the decolorization and recovery ratios of polysaccharides extracted from mulberry leaves using various resins. The experimental data reveal that resins AB-8 and D152 have higher recovery ratios which are above 85%, whereas resins 732, D152, and D113 demonstrate superior decolorization ratios which are nearly 60%. Based on these findings, resin D152 was selected for further polysaccharide separation in subsequent experiments.

#### 2.3.2. Influence of Column Liquid Concentration

**Figure 4:**
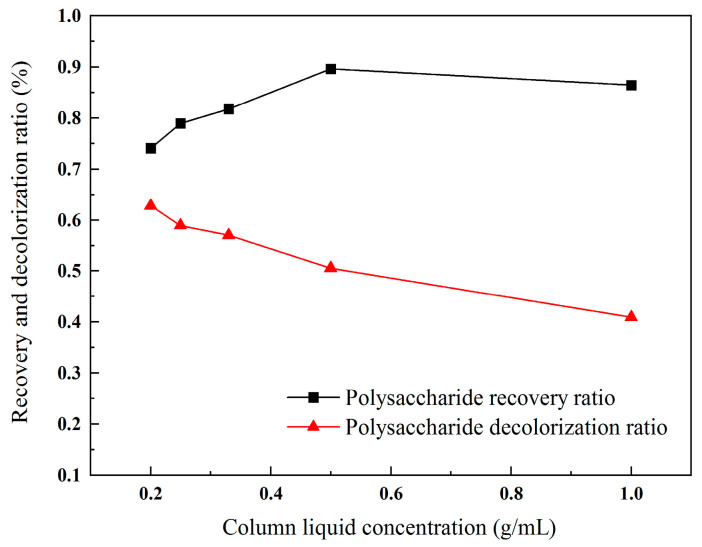
Recovery ratio and decolorization ratio of polysaccharides with different concentrations of extracting solution.

When the total amount of polysaccharides remained constant, an increase in the concentration of the solution corresponded to a decrease in the volume of the solution and a reduction in the purification time required for the resin [26]. Concurrently, during the decolorization process, the resin exhibited enhanced adsorption efficiency for fine particles within the solution [27]. However, the resin’s adsorption capacity is finite. Consequently, higher concentrations of the polysaccharide extract led to increased recovery ratios, although the decolorization efficacy tended to decrease. As shown in Figure 4, with a continuous increase in the column liquid concentration, the decolorization ratio of the polysaccharides shows a gradual downward trend, while the recovery ratio first increases and then begins to decrease slowly when the concentration exceeds 0.5 g/mL. Therefore, the optimal column loading concentration is 0.5 g/mL.

#### 2.3.3. Influence of Flow Rate

**Figure 5:**
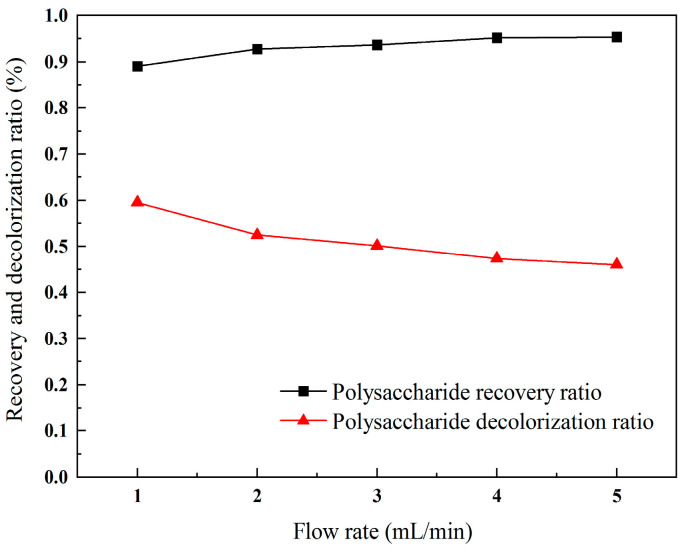
Recovery ratio and decolorization ratio of polysaccharides with different flow rates.

The flow rate governs the contact time between the resin and the polysaccharide solution. An excessively rapid flow rate results in inadequate adsorption, leading to incomplete adsorption of both polysaccharides and pigments. While a slight increase in the flow rate may marginally enhance the recovery ratio of polysaccharides, it concurrently deteriorates the decolorization efficiency. As shown in Figure 5, with a continuous increase in the flow rate, the recovery ratio of the polysaccharides keeps rising, while the decolorization ratio starts to decrease from 60%, showing a continuous downward trend. Therefore, the optimal flow rate for the decolorization process was found to be 1 mL/min.

### 2.4. Purification and Separation of Polysaccharides by Activated Carbon

#### 2.4.1. Effect of Activated Carbon Dose on Polysaccharide Purification

**Figure 6:**
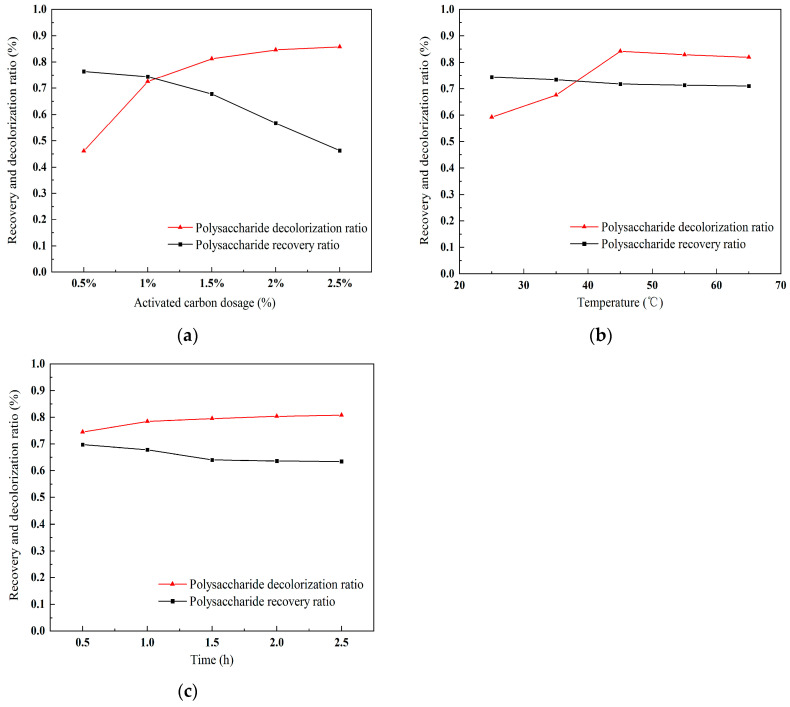
Effects on purification of polysaccharides of activated carbon. (**a**) Effect of activated carbon dose on polysaccharide purification; (**b**) effect of temperature on polysaccharide purification; (**c**) effect of time on polysaccharide purification.

Activated carbon adsorption, which relies on van der Waals and electrostatic forces to capture pigments, is also a commonly used method for polysaccharide discoloration. Figure 6a illustrates the impact of activated carbon dosage on the purification of polysaccharides. As indicated in the figure, an increase in the activated carbon dosage corresponds to a continuously improving decolorization ratio; however, this is accompanied by a gradual increase in the loss of polysaccharides. The recovery ratio of polysaccharides decreases with the escalating dose of activated carbon. Consequently, while the decolorization ratio significantly improves with the addition of activated carbon, the recovery ratio of polysaccharides also experiences a marked decline. Therefore, it is advisable to avoid using an excessively high dose of activated carbon. Given that at a dosage of 2.0%, the loss of polysaccharides exceeds half, it is appropriate to choose 1% as the optimal dosage, as it provides a relatively high recovery ratio and decolorization ratio.

#### 2.4.2. Effect of Temperature on Polysaccharide Purification

An increase in temperature enhances molecular movement, thereby facilitating the adsorption process of activated carbon on substance molecules. Figure 6b presents the results for the effect of temperature on the purification of polysaccharides. The figure shows that the decolorization ratio gradually increases with rising temperature. The optimal decolorization effect is achieved at a temperature of 45 °C. The loss rate of polysaccharides does not exhibit significant changes with increasing temperature. Therefore, the optimal temperature for the purification of polysaccharides using activated carbon is determined to be 45 °C, as it provides favorable outcomes for both decolorization and recovery.

#### 2.4.3. The Influence of Time on Polysaccharide Purification

Figure 6c displays the results for the time-dependent effects on the purification of polysaccharides using activated carbon. The figure indicates that the influence of time on the adsorption of pigments and the purification of polysaccharides by activated carbon is minimal. With increasing time, the rate of color removal and the recovery ratio of polysaccharides remained relatively balanced. There was a slight increase in the rate of color removal and a slight decrease in the recovery ratio of polysaccharides as the time extended from 0.5 to 1 h. Beyond 1 h, the adsorption process reached equilibrium, with the loss of color and polysaccharides stabilizing. Consequently, the optimal adsorption time was determined to be 1 h.

In summary, the use of activated carbon adsorption is an optional method for polysaccharide decolorization. The optimal conditions for activated carbon decolorization obtained in this study are as follows: a dosage of 1%, an adsorption temperature of 45 °C, and an adsorption time of 1 h. However, the method of activated carbon adsorption has poor selectivity between pigments and polysaccharides. This lack of specificity may result in the loss of polysaccharides.

### 2.5. Analysis of Polysaccharides Extracted by Ultrasonic-Assisted Extraction

#### 2.5.1. Thin-Layer Chromatography Analysis

The purified mulberry leaf polysaccharides were subjected to hydrolysis, following which their compositional analysis was conducted via thin-layer chromatography (TLC) [28,29]. Through a comparison of the Rf values of the extract with those of standard monosaccharides, it was determined that the polysaccharides from mulberry leaves are primarily composed of rhamnose, xylose, and arabinose, as presented in Table 5.

#### 2.5.2. FT-IR Spectra Analysis

**Figure 7:**
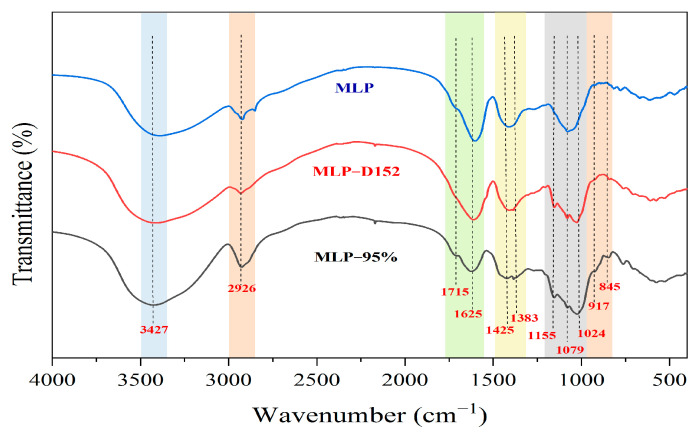
Comparative FT-IR spectra of crude extract (MLP), resin D152-purified (MLP-D152), and 95% purity standard (MLP-95%) MLPs.

Figure 7 comparatively analyzes the Fourier-transform infrared spectroscopy (FTIR) characteristics of the crude extract of MLPs, the resin D152-purified sample (MLP-D152), and the 95% purity standard product (MLP-95%). All three exhibited typical polysaccharide structural signals, including a hydroxyl (-OH) stretching vibration peak at 3427 cm^−1^, a C-H bond stretching vibration peak at 2926 cm^−1^, a stretching vibration peak of esterified carboxyl at 1715 cm^−1^, and a C-O bond stretching vibration peak at 1425 cm^−1^. The absorption peak intensity at 1625 cm^−1^ of the purified sample (MLP-D152) and the standard product (MLP-95%) was significantly lower than that of the crude extract (MLP), indicating a weakened C=O vibration of the amide I band [30], which suggested that the resin D152 purification process effectively reduced the trace protein residue in the sample and was consistent with the spectral trend of the standard product. The region of high absorbance between 1155 and 1024 cm^−1^ represents the characteristic absorption peaks of polysaccharides [31,32]. The symmetrical vibration peaks of C-O-C or C-O-H in the range of 1155 to 1024 cm^−1^ were clearly visible [33,34], confirming the presence of pyran rings. The two absorption bands at 917 cm^−1^ and 845 cm^−1^ indicated the existence of β- and α-configurations in the sugar molecules [11,35], and the purified mulberry leaf polysaccharides were highly consistent with the standard product. In conclusion, the resin D152 purification process effectively removed protein impurities while completely retaining the basic structural units such as glycosidic bonds and sugar rings of MLP, further verifying the effectiveness of the purification process.

#### 2.5.3. SEM Analysis

**Figure 8:**
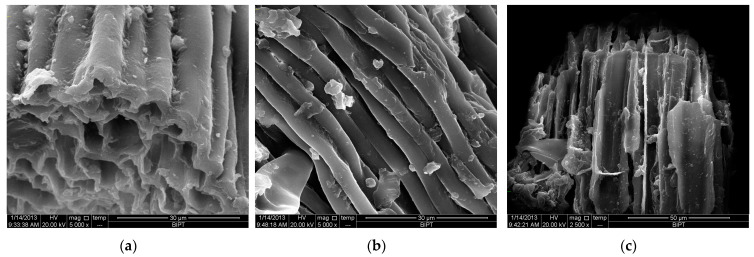
SEM images of solid surface of mulberry leaf residue for different extraction processes. (**a**) SEM image of solid surface of raw mulberry leaf; (**b**) SEM image of solid surface of mulberry leaf residue after traditional solvent extraction; (**c**) SEM image of solid surface of mulberry leaf residue after ultrasonic extraction.

The microstructural alterations in mulberry leaf tissues following extraction were examined using scanning electron microscopy (SEM). Figure 8 illustrates that the extraction methods resulted in distinct changes to the physical structure of the tissues. Tissues subjected to conventional solvent extraction (CSE) preserve nearly intact cell walls (Figure 8b). In contrast, tissues treated with ultrasound-assisted extraction (UAE) (Figure 8c) exhibit substantial damage. UAE had a more pronounced effect on the cell walls compared to CSE. The increased presence of spongy structures and debris suggests that ultrasonic vibration altered the internal architecture of the material, while the cavitation effect enhanced mass transfer rates, thereby improving extraction efficiency [36,37,38]. During the CSE process, the heated solvent gradually permeated the cell walls, solubilizing and extracting the target compounds with minimal damage to the tissue microstructure.

## 3. Materials and Methods

### 3.1. Materials and Chemicals

The mulberry leaves used in this study were provided by the Royal Lin Ancient Mulberry Field located in Daxing, Beijing. The mulberry leaves were processed into a fine powder. Standard monosaccharides (glucose, sorbitol, rhamnose, arabinose, xylose, mannose, and galactose) were purchased from Sinopharm. Macroporous resins including AB-8, 201, D101, D152, JK206, JK008, D113, and 732 were purchased from DongHong Chemical Company (Zibo, China). All other chemicals employed were of analytical grade.

### 3.2. Preparation of Mulberry Leaves

The raw material was processed as follows: mulberry leaves were immersed in 95% ethanol overnight, followed by treatment with 80% ethanol at 50 °C for 2 h to defat and remove some colored materials and lipid-soluble impurities. The ethanolic extract was then separated using filtration. After mulberry leaves were defatted with ethanol, the residue was air-dried at room temperature to constant weight, ground into powder (60 mesh), and stored in a dryer for later use.

### 3.3. Extraction of MLPs with CSE

Conventional solvent extraction was carried out in an HH-6 water bath (Guohua Wiring Company, Shanghai, China). A total of 10.0 g of the ground powder was mixed with distilled water in a round-bottomed flask. Extractions were carried out with the conditions of a liquid-to-material ratio of 16:1 mL/g, extraction time of 58 min, and extraction temperature of 65 °C. The polysaccharide solution obtained by filtration was concentrated using a rotary evaporator and precipitated with four times the volume of 95% (*v*/*v*) ethanol at 4 °C for 48 h. The precipitates were collected by centrifugation (6000 r/min, 30 min) and dried to constant weight at room temperature.

### 3.4. Extraction of MLPs with UAE

Several parameters were investigated to analyze their effects on the extraction process. The preliminary range of values for the extraction parameters was determined through a seriatim–factorial experiment. The parameters included the liquid-to-material ratio (8:1, 10:1, 12:1, 14:1, 16:1 mL/g), extraction time (20, 40, 60, 80, 100 min), temperature (20, 30, 40, 50, 60 °C), and ultrasonic power (300, 350, 400, 450, 500 W). Each experiment was conducted in triplicate. The range of these parameters was selected based on the yield of polysaccharides. All data obtained were utilized for subsequent experimental designs.

### 3.5. Response Surface Methodology Experimental Designs

Response surface methodology (RSM) is an empirical statistical technique that employs multiple regression analysis using quantitative data obtained from well-designed experiments to solve multivariate equations simultaneously [39,40,41]. A Box–Behnken design (BBD), consisting of a total of 29 experiments, was utilized to optimize the extraction conditions for the polysaccharide extraction process from mulberry leaves. The range and levels of the individual variables are provided in Table 6, with −1 representing the low level, 1 the high level, and 0 the central level. The yield of polysaccharides extracted from mulberry leaves served as the response variable.

### 3.6. Purification of MLPs

#### 3.6.1. Pretreatment of Macroporous Resins

Macroporous resins necessitate rigorous pretreatment before use. Precisely weigh a suitable amount of resin and immerse it in 95% ethanol at a volume twice that of the resin for an overnight period. Thereafter, wash the resin with deionized water until any residual alcohol flavor is absent. Following this, immerse the resin in a 4% NaOH solution, with a volume twice that of the column, for a duration of two hours, and subsequently, rinse with deionized water until a neutral pH is obtained. Subsequently, treat the resin with a 4% HCl solution, at a volume twice that of the column, for an additional two hours, followed by another rinse with deionized water until neutrality is confirmed. After the completion of these steps, the resin will be ready for use.

#### 3.6.2. Static Adsorption Experiment with Macroporous Resin

This study involved the selection of eight types of resins with varying specifications, including Jk008, 732, AB-8, D101, D307, JK206, D202, and D152, to determine the most suitable resin, with polysaccharide recovery and decolorization ratios as the evaluation criteria. Conical flasks were labeled, and a measured amount of each macroporous resin was added to the respective flask. To each flask, 25 mL of the crude polysaccharide extraction solution was introduced, and the mixtures were agitated at room temperature. Following a 24 h period, the absorbance of the extraction solution was measured at 420 nm and 620 nm, respectively. The absorbance at 620 nm was quantified using the anthrone–sulfuric acid method. Based on the polysaccharide recovery and decolorization ratios, the optimal resin was selected. The polysaccharide recovery ratio refers to the ratio of the polysaccharide content in the solution after separation and purification to that before purification, and the decolorization ratio refers to the rate of change in the absorbance of the solution before and after decolorization.

#### 3.6.3. Dynamic Adsorption Experiment with Macroporous Resin

The optimum amount of pretreated resin was loaded into a wet-packed column (25 × 500 mm), and the polysaccharide extract was concentrated to 100 mL within the column. Aliquots of 10 mL were collected sequentially, and for each fraction, the decolorization ratio (absorbance at A420) and the polysaccharide concentration (absorbance at A620, determined by the anthrone–sulfuric acid method) were measured.

The concentration and flow rate of the feed solution are critical factors influencing the adsorption and purification of polysaccharides using macroporous resin. Polysaccharide extracts with varying initial concentrations (100 mL each) were passed through the resin column, and the effluent was collected. The eluate was then quantitatively analyzed using the anthrone–sulfuric acid method to determine the polysaccharide recovery rate. The effect of the polysaccharide concentration in the feed solution on the purification process was examined, with the aim of achieving a target polysaccharide recovery rate.

Equal amounts of polysaccharide extract (100 mL each) were passed through the resin column at varying flow rates. The effluent was collected, and the elution fractions were combined and quantitatively analyzed using the anthrone–sulfuric acid method to determine the polysaccharide recovery rate. The impact of different feed solution concentrations on the polysaccharide recovery rate was investigated, with the recovery rate serving as the evaluation criterion.

#### 3.6.4. Purification of Polysaccharide by Activated Carbon

Trace amounts of Zn^2+^, Fe^3+^, Ca^2+^, and phosphate are present in natural activated carbon. The presence of these impurities endows the activated carbon with a charge, which can influence its adsorption efficiency. The pretreatment procedure involved taking a specific quantity of activated carbon and soaking it in 15% acetic acid for 1.5 to 2 h. Subsequently, the activated carbon was washed with deionized water until neutrality was achieved. Following this, the activated carbon was dried in an oven at 120 °C for 4 h to complete the activation process.

The purification process of activated carbon involves the following steps: 50 mL polysaccharide extract is placed in a conical flask, to which pretreated granular activated carbon is added. The mixture is then stirred using a magnetic stirrer at a specific temperature. After stirring, the mixture is allowed to stand, and the supernatant is separated and analyzed for absorbance at 420 nm and 620 nm to determine the polysaccharide recovery ratio and decolorization ratio, respectively. The variables investigated in the purification process include the dosage of activated carbon, temperature, and time (Table 7).

### 3.7. Analytical Methods

#### 3.7.1. Quantitative Determination of Polysaccharides

Following hydrolysis in concentrated sulfuric acid, the polysaccharide is further dehydrated to form aldose derivatives, which can react with anthrone to produce a colored compound. The determination of MLPs was carried out employing the phenol–sulfuric acid method with glucose serving as the standard as follows [42]: draw 1 mL of the polysaccharide sample into a test tube and cool it in an ice bath. Add 4 mL of the anthrone reagent (prepared by dissolving 0.2 g of anthrone in 100 mL of concentrated sulfuric acid) to the sample. Subsequently, place the test tube in a boiling water bath for 10 min, and then cool it under running water. Afterward, allow the solution to stand at room temperature for 20 min. The absorbance of the colored solution is then measured at 620 nm.

#### 3.7.2. Fourier-Transform Infrared (FT-IR) Spectra

The Fourier-transform infrared (FT-IR) spectroscopy of the polysaccharides was performed using the potassium bromide (KBr) pellet method on a VERTEX 70 Fourier Transform Infrared Spectrometer (Bruker, Karlsruhe, Germany). The spectra were recorded in the range of 400–4000 cm^−1^.

#### 3.7.3. Thin-Layer Chromatography

The monosaccharides were released from MLPs by acid hydrolysis with trifluoroacetic acid (2 M) at 120 °C for 1.5 h [43,44], and then were analyzed by thin-layer chromatography. Preparation of the sample: glucose, arabinose, galactose, rhamnose, xylose, mannose, and various monosaccharides were, respectively, weighed to 10 mg and dissolved in deionized water to prepare a single control solution and its mixture. Development and color development: each monosaccharide sample was sequentially sampled on the silica gel G plate by a capillary tube and placed into the chromatographic cylinder for chromatography. The developing agent was ethyl acetate/acetic acid/water (3:1:3). When the solvent front was close to the top of the silicone sheet, within 1 cm, the silicone sheet was taken out and dried at 100 °C for 5 min. The color development agent (10% concentrated sulfuric acid) was sprayed on the silica gel plate, and it was heated in an oven at 100 °C for 10 min [45].

#### 3.7.4. Scanning Electron Microscopy (SEM)

The raw material, as well as the residues from hot water extraction and ultrasonic-assisted extraction, was analyzed using an FEI Quanta 400 FEG scanning electron microscope (Thermo Fisher, Brno, Czech Republic) under high vacuum conditions at an accelerating voltage of 20.0 kV.

## 4. Conclusions

This study focused on optimizing multiple extraction parameters to maximize the yield of polysaccharides from mulberry leaves, utilizing ultrasonic-assisted extraction (UAE). Under the ultrasonic-assisted extraction conditions (1:16 mL/g, 58 min, 500 W, 65 °C) obtained by response surface optimization, the extraction yield of polysaccharides was 14.47%, which is significantly higher than the yields reported for traditional solvent and microwave extraction methods. Scanning electron microscopy (SEM) was used to investigate the changes in mulberry leaves before and after ultrasonic-assisted and traditional solvent extraction. The results showed that ultrasonic treatment could significantly disrupt the cell structure of mulberry leaves, which helped to improve the extraction efficiency of mulberry leaf polysaccharides.

This study also investigated the separation and purification effects of mulberry leaf polysaccharides using macroporous resin and activated carbon. The results indicate that activated carbon has a good decolorization effect but results in a significant loss of polysaccharides. Although the decolorization effect of the macroporous resin method was slightly lower than that of activated carbon, it had a higher recovery ratio of polysaccharides. Eight different types of resin were assessed in the separation and purification process, with the D152 resin being identified as the most suitable. The optimal conditions for the purification process were found to be a sample solution concentration of 0.5 g/mL and an optimal flow rate of 1 mL/min. Infrared spectroscopy was used to investigate the changes in MLPs before and after decolorization with the D152 resin, and the results also confirmed that the D152 resin has good decolorization and deproteinization effects.

In recent years, there has been a significant increase in interest in plant-derived polysaccharides within the food, pharmaceutical, and biomedical sectors. This heightened interest is due to their inherent hydrophilic properties, stability, safety profile, non-toxicity, and biodegradability, which collectively classify them as green and eco-friendly dual-purpose pharmaceutical and food substances. Currently, the extraction of MLPs mostly remains at the laboratory research level and cannot be regarded as a true extraction technology. Therefore, it is necessary to study extraction technology and integrate the technical system according to the needs of large-scale production, at least reaching the pilot scale. In short, further research on the extraction, separation, and purification, structural characterization, biological activity, mechanism of action, and quality control of MLPs is very necessary, in order to provide a scientific basis for their comprehensive development and utilization.

## Figures and Tables

**Table 1:** Box–Behnken experimental design with the independent variables.

	X_1_	X_2_	X_3_	X_4_	Response (Y, Yield, %)
1	0	−1	−1	0	8.49
2	−1	0	−1	0	7.85
3	0	0	−1	−1	5.49
4	0	0	−1	1	12.94
5	1	0	−1	0	8.05
6	0	1	−1	0	7.85
7	0	−1	1	0	10.13
8	−1	0	1	0	8.15
9	0	0	1	−1	6.81
10	0	0	1	1	13.45
11	1	0	1	0	12.08
12	0	1	1	0	11.65
13	−1	−1	0	0	7.12
14	0	−1	0	−1	7.01
15	0	−1	0	1	12.79
16	1	−1	0	0	11.49
17	−1	0	0	−1	6.13
18	−1	0	0	1	10.65
19	1	0	0	−1	5.95
20	1	0	0	1	14.03
21	−1	1	0	0	9.16
22	0	1	0	−1	7.16
23	0	1	0	1	13.42
24	1	1	0	0	10.18
25	0	0	0	0	11.26
26	0	0	0	0	11.47
27	0	0	0	0	11.35
28	0	0	0	0	11.53
29	0	0	0	0	11.42

**Table 2:** Regression coefficients of the predicted quadratic polynomial model.

Parameter	Estimate	Error	F-Value	*p*-Value	Significance
X_1_	1.06	0.14	59.69	<0.0001	***
X_2_	0.2	0.14	2.11	0.1686	--
X_3_	0.97	0.14	49.64	<0.0001	***
X_4_	3.23	0.14	553.38	<0.0001	***
X_1_X_2_	−0.84	0.24	12.42	0.0034	**
X_1_X_3_	0.93	0.24	15.40	0.0015	**
X_1_X_4_	0.89	0.24	14.03	0.0022	**
X_2_X_3_	0.54	0.24	5.16	0.0394	*
X_2_X_4_	0.12	0.24	0.25	0.6214	--
X_3_X_4_	−0.20	0.24	0.73	0.4085	--
X_1_^2^	−1.35	0.19	52.28	<0.0001	***
X_2_^2^	−0.65	0.19	12.06	0.0037	**
X_3_^2^	−1.09	0.19	33.91	<0.0001	***
X_4_^2^	−0.73	0.19	15.11	0.0016	**
Model	11.41	0.21	56.47	<0.0001	***

* Significant at 0.05 level; ** significant at 0.01 level; *** significant at 0.001 level.

**Table 3:** Analysis of variables for the fitted quadratic polynomial model of extraction.

Mean	C.V.%	PRESS	R-Squared	Adj R-Squared	Pred R-Squared	Adeq Precision
9.83	4.84	18.03	0.9862	0.9562	0.9008	27.242

**Table 4:** Predicted and experimental values of the response in optimum and modified conditions.

	Ratio of Water to Raw Material (mL/g)	Extraction Time (min)	Ultrasonic Power (W)	Extraction Temperature (°C)	Yield (%)
Prediction	16.1:1	58.2	484.5	65.1	15.17%
Actual	16:1	58	500	65	14.47 ± 0.76%

**Table 5:** The Rf values determined by TLC.

Monosaccharides	Rhamnose	Xylose	Arabinose
Rf values	0.63	0.59	0.66

**Table 6:** Levels of the variables of the Box–Behnken design.

Variables	Symbol	Experimental Value		
		Low, −1	Zero, 0	High, 1
The ratio of liquid to material (mL/g)	X_1_	12:1	14:1	16:1
Time (min)	X_2_	40	60	80
Power (W)	X_3_	400	450	500
Temperature (°C)	X_4_	30	40	50

**Table 7:** The factors and their values.

Factors	Activated Carbon Dosage/%	Temperature/°C	Time/h
Values	0.5, 1.0, 1.5, 2, 2.5	25, 35, 45, 55, 65	0.5, 1, 1.5, 2, 2.5

## Data Availability

Data are contained within the article.

## References

[1] Ma J.K., Yan X.L., Xu H.Z., Pan L.C., Zhai X.L., Xue Y., Chen Y.J., Liu H.P., Zhao M., Luo L. (2024). Effects of mulberry leaf extract on growth, digestion, liver lipid metabolism and hypoglycaemic ability in mandarin fish (Siniperca chuatsi). Aquacult. Rep..

[2] Singh A., Dar M.Y., Sharma A., Sharma S., Shrivastava S., Shukla S. (2017). Therapeutic efficacy of Morus alba L. against N-nitrosodiethylamine induced subchronic hepatic ailment in rats. Toxicol. Environ. Health Sci..

[3] Zhong S., Yang Y., Huo J.X., Sun Y.Q., Ren N., Lu Q.H., Li D., Zhan P.F., Wu W.J., Chen H.Z. (2023). Dissection of gut microbiota and metabolites reveals the hypolipidemic effect of green mulberry leaf tea / black mulberry leaf tea in mice. J. Funct. Foods.

[4] Akbari Aghdam M., Pagan A., Garcia-Esta J., Atucha N.M. (2025). Evaluation of the Effects of Mulberry Leaf Extracts Morus alba L. on Cardiovascular, Renal, and Platelet Function in Experimental Arterial Hypertension. Nutrients.

[5] Chen R., Zhou X., Deng Q., Yang M., Li S., Zhang Q., Sun Y., Chen H. (2024). Extraction, structural characterization and biological activities of polysaccharides from mulberry leaves: A review. Int. J. Biol. Macromol..

[6] Wang H.L., Huang G.L., Zhang X.X. (2025). Analysis and properties of polysaccharides extracted from *Brassica oleracea* L. var. capitata L. by hot water extraction/ultrasonic-synergistic enzymatic method. Ultrason. Sonochem..

[7] Zhang Y.H., Song H.Z., Lu J., Wang F., Wang L.F., Xiong L., Shen X.C. (2025). Ultrasound-assisted extraction, purification, structural characterization, and hypoglycemic activities of a polysaccharide from *Momordica charantia* L.. Int. J. Biol. Macromol..

[8] Meng L., Chen Y., Zheng Z.J., Wang L., Xu Y.H., Li X.J., Xiao Z.J., Tang Z., Wang Z.S. (2024). Ultrasound-Assisted Extraction of Paeonol from Moutan Cortex: Purification and Component Identification of Extract. Molecules.

[9] Leichtweis M.G., Molina A.K., Petropoulos S.A., Carocho M., Pires T.C.S.P., Dias M.I., Calhelha R., Oliveira M.B.P.P., Pereira C., Barros L. (2023). Valorization of Pumpkin Peel as a Source of Bioactive Compounds: Optimization of Heat- and Ultrasound-Assisted Extraction. Molecules.

[10] Thajudeen K.Y., Asiri Y.I., Salam S., Thorakkattil S.A., Rahamathulla M., Uoorakkottil I. (2022). A Box–Behnken Extraction Design and Hepatoprotective Effect of Isolated Eupalitin-3-O-β-D-Galactopyranoside from Boerhavia diffusa Linn. Molecules.

[11] Chen G., Sun M., Chen K., Wang L., Sun J. (2024). Ultrasonic-assisted decoloration of polysaccharides from seedless chestnut rose (rosa sterilis) fruit: Insight into the impact of different macroporous resins on its structural characterization and in vitro hypoglycemic activity. Foods.

[12] Kirankumar P.S., Tian L.L., Li H., Johnston C.T., Boyd S.A., Teppen B.J. (2025). Accelerated solvent extraction of dioxins sequestered in activated carbon: A response surface methodology-based optimization. Chemosphere.

[13] Wang Z.M., Wu S.S., Wang J.Y., Yang C., Wang Y., Hu Z., Cai W., Liu L.L. (2024). Optimization of Polysaccharide Extraction from Polygonatum cyrtonema Hua by Freeze–Thaw Method Using Response Surface Methodology. Molecules.

[14] Solhi L., Sun H.S., Daswani S.H., Shojania S., Springate C.M.K., Brumer H. (2021). Controlled sulfation of mixed-linkage glucan by Response Surface Methodology for the development of biologically applicable polysaccharides. Carbohydr. Polym..

[15] Zhu B.E., Li C., Yao Z., Xu H., Ning L.M. (2025). Efficient degradation of the polysaccharide extracted from Enteromorpha prolifera by using a novel polysaccharide lyase family 28 enzyme with high activity. Food Chem..

[16] Jiang S., Wang Q., Wang Z., Borjigin G., Sun J., Zhao Y., Li Q., Shi X., Shah S.F.A., Wang X. (2024). Ultrasound-assisted polysaccharide extraction from Fritillaria ussuriensis Maxim. and its structural characterization, antioxidant and immunological activity. Ultrason. Sonochem..

[17] Lai J.J., Zhou P., Li X.Z., Lu Y., Wang Y.Q., Yuan H., Yang Y.H. (2025). Ultrasound-assisted deep eutectic solvent extraction of flavonol glycosides from Ginkgo biloba: Optimization of efficiency and mechanism. Ultrason. Sonochem..

[18] Koda S., Kimura T., Kondo T., Mitome H. (2003). A standard method to calibrate sonochemical efficiency of an individual reaction system. Ultrason. Sonochem.

[19] Zhao S., Kwok K.C., Liang H. (2007). Investigation on ultrasound assisted extraction of saikosaponins from radix bupleuri. Sep. Purif. Technol..

[20] Myers R.H., Montgomery D.C. (1995). Response Surface Methodology: Process and Product Optimization Using Designed Experiments.

[21] Zhao H., Yang J., Zeng J., Zhou B., Yang M., Yang X., Sun R. (2025). Development of subcritical water extraction for areca alkaloids and its influence on the structure of areca nut husk. Molecules.

[22] Chen Q., Zhang W., Wang Y., Cai W., Ni Q., Jiang C., Li J., Shen C. (2025). Genetic Algorithm-Back Propagation Neural Network Model- and Response Surface Methodology-Based Optimization of Polysaccharide Extraction from Cinnamomum cassia Presl, Isolation, Purification and Bioactivities. Foods.

[23] Samavati V., Yarmand M.S. (2013). Statistical modeling of process parameters for the recovery of polysaccharide from morus alba leaf. Carbohydr. Polym..

[24] Thirugnanasambandham K., Sivakumar V., Maran J.P. (2014). Microwave-assisted extraction of polysaccharides from mulberry leaves. Int. J. Biol. Macromol..

[25] Ying Z., Han X., Li J. (2011). Ultrasound-assisted extraction of polysaccharides from mulberry leaves. Food Chem..

[26] Zhang X., Shi C., Wang Z., Dai J., Guan C., Sheng J., Tao L., Tian Y. (2025). Separation, Purification, Structural Characterization, and In Vitro Hypoglycemic Activity of Polysaccharides from Panax notoginseng Leaves. Molecules.

[27] Meng Q., Wang Y., Rong M., Xing H., Chi R.-A., Chen C., Liu H., Yang L. (2024). Efficient Adsorption and Separation of Glyphosate in Aqueous Solution by Amino-Functionalized Poly(Glycidyl Methacrylate). Chem. Eng. J..

[28] Wang Y., Huang G. (2025). Preparation, Structure and Properties of Litchi Pericarp Polysaccharide. Sci. Rep..

[29] Bian Z., Ding J., Zhang X., Zhang J., Zhang C., Shang G., Zhu L., Zhang Y., Liu Q., Liu Y. (2024). Multiple Fingerprinting and Chromatography-Immunoreactivity Relationship of Polysaccharides from Dioscorea Opposite Thunb. Food Chem..

[30] Kawasaki T., Fujioka J., Imai T., Torigoe K., Tsukiyama K. (2014). Mid-infrared free-electron laser tuned to the amide I band for converting insoluble amyloid-like protein fibrils into the soluble monomeric form. Lasers Med. Sci..

[31] Zhuan N., Lulu C., Guangxia L., Hui L., Yanping L., Jianlong M., Jianbao D., Jin Y. (2024). A Method for the Quantitative Analysis of Lycium Barbarum Polysaccharides (LBPs) Using Fourier-Transform Infrared Spectroscopy (FTIR): From Theoretical Computation to Experimental Application. Spectrochim. Acta Part A.

[32] Yang H., Lei C., Li D., Zhang N., Lang Y., Wu L., Wang M., Tian H., Li C. (2025). A Comparative Investigation on the Extraction-Function Relationship of Polysaccharides Derived from Moringa Oleifera Seeds in Terms of Antioxidant Capacity. Food Chem..

[33] Kacuráková M., Capek P., Sasinková V., Wellner N., Ebringerová A. (2000). FT-IR study of plant cell wall model compounds: Pectic polysaccharides and hemicelluloses. Carbohydr. Polym..

[34] Yang C.X., He N., Ling X.P., Zhang C.X., Yao C.Y., Wang Z.Y., Li Q.B. (2008). The Isolation and Characterization of Polysaccharides from Longan Pulp. Sep. Purif. Technol..

[35] Chen J., Zhou M., Liu M., Bi J. (2022). Physicochemical, rheological properties and in vitro hypoglycemic activities of polysaccharide fractions from peach gum. Carbohydr. Polym..

[36] Cui R., Zhu F. (2021). Ultrasound modified polysaccharides: A review of structure, physicochemical properties, biological activities and food applications. Trends Food Sci. Technol..

[37] Du B., Jeepipalli S.P.K., Xu B. (2022). Critical review on alterations in physiochemical properties and molecular structure of natural polysaccharides upon ultrasonication. Ultrason. Sonochem..

[38] Wei Q., Zhang Y.H. (2023). Ultrasound-assisted polysaccharide extraction from Cercis chinensis and properites, antioxidant activity of polysaccharide. Ultrason. Sonochem..

[39] Ng Y.V., Ali M.K.M., Wan Ishak W.R. (2025). Optimization of aqueous extraction conditions for bioactive compounds and antioxidant properties of overripe banana (Musa acuminata) using response surface methodology. J. Agric. Food Res..

[40] Atanacković Krstonošić M., Sazdanić D., Mikulić M., Ćirin D., Milutinov J., Krstonošić V. (2025). Optimization of Surfactant-Mediated Green Extraction o f Phenolic Compounds from Grape Pomace Using Response Surface Methodology. Int. J. Mol. Sci..

[41] Robles-Apodaca S.M., González-Vega R.I., Ruíz-Cruz S., Estrada-Alvarado M.I., Cira-Chávez L.A., Márquez-Ríos E., Del-Toro-Sánchez C.L., Ornelas-Paz J.d.J., Suárez-Jiménez G.M., Ocaño-Higuera V.M. (2024). Optimization of Extraction Process for Improving Polyphenols and Antioxidant Activity from Papaya Seeds (Carica papaya L.) Using Response Surface Methodology. Processes.

[42] Wang C.Z., Zhang H.Y., Li W.J., Ye J.Z. (2015). Chemical Constituents and Structural Characterization of Polysaccharides from Four Typical Bamboo Species Leaves. Molecules.

[43] Ji X., Heng Y., Tian J., Zhang S., Shi M. (2021). Structural characterization of polysaccharide from jujube (ziziphus jujuba mill.) fruit. Chem. Biol. Technol. Agric..

[44] Ji X., Guo J., Tian J., Ma K., Liu Y. (2023). Research progress on degradation methods and product properties of plant polysaccharides. J. Light. Ind..

[45] Lin J.K., Wu S.S. (1987). Synthesis of dabsylhydrazine and its use in the chromatographic determination of monosaccharides by thin-layer and high-performance liquid chromatography. Anal. Chem..

